# Fractionated Marine Invertebrate Extract Libraries for Drug Discovery

**DOI:** 10.3390/molecules13061372

**Published:** 2008-06-19

**Authors:** Tim S. Bugni, Mary Kay Harper, Malcolm W.B. McCulloch, Jason Reppart, Chris M. Ireland

**Affiliations:** Department of Medicinal Chemistry, University of Utah, 30 S. 2000 E. RM 307, Salt Lake City, UT 84112, USA

**Keywords:** Sponge, Tunicate, Prefractionation, HP20SS, Marine Natural Products, LCMS

## Abstract

The high-throughput screening and drug discovery paradigm has necessitated a change in preparation of natural product samples for screening programs. In an attempt to improve the quality of marine natural products samples for screening, several fractionation strategies were investigated. The final method used HP20SS as a solid support to effectively desalt extracts and fractionate the organic components. Additionally, methods to integrate an automated LCMS fractionation approach to shorten discovery time lines have been implemented.

## Introduction

Marine invertebrates have been a rich source of chemical diversity and pharmaceutical leads. However, it has been estimated that only a small percentage of the total number of estimated species in the marine environment have been investigated [1]. Data extracted from the NCI preclinical antitumor drug discovery screen showed that sponges (phylum Porifera) exhibited more cytotoxic extracts compared to plants and other marine invertebrates [1]. Tunicates (phylum Chordata) and bryozoans (phylum Bryozoa) also yielded high numbers of cytotoxic extracts. Since then, investigation of these groups has led to the discovery of numerous pharmaceutical leads, particularly in the anticancer drug discovery area [2,3]. Tunicates in particular have been a prolific source of cytotoxic natural products. Last year, the first anticancer agent from a marine invertebrate, Trabectedin^®^ (ecteinascidin-743, ET743) [4,5] was approved in Europe for advanced soft tissue sarcoma and has been marketed by PharmaMar. ET743 was first isolated from the tunicate *Ecteinascidia turbinata*. Another tunicate derived natural product, Aplidin^®^ (dehydrodidemnin B) [6,7], is currently undergoing phase II clinical trials, sponsored by PharmaMar. Numerous anticancer agents from marine invertebrates are in clinical trials [8] or are in preclinical development [2]. On the basis of the successes outline above, our natural products discovery program has largely focused on the discovery of anticancer agents from marine sponges and tunicates [9].

Marine natural product extracts present several problems with respect to modern drug discovery programs. The first and foremost problem encountered with marine invertebrate extracts from sponges and tunicates is the presence of large quantities of inorganic salts. Additionally, the chemical diversity found in one sponge may represent several different classes of bioactive molecules that exhibit different and sometimes opposing pharmacological activities. In many cases, the presence of a major non-selective compound can mask the activity of minor selective compounds. Minor compounds in many cases are present in crude extracts at concentrations that are below detection thresholds. From a discovery standpoint, these problems can be addressed to a certain point through the use of prefractionation strategies [10,11,12,13,14].

From a screening standpoint, complex natural product mixtures can present numerous problems in high-throughput screens [10,12,13,14,15,16]. As part of a National Cooperative Drug Discovery Group (NCDDG), we observed a low hit rate for natural product extracts in high-throughput screening (HTS). Two major problems were observed. First, active components were many times 0.1% and sometimes less than 0.01% of the crude extract, which resulted in many components being below screening thresholds. Second, active components were being masked by other major metabolites.

## Results and Discussion

In order to develop methods that addressed the issues with screening mixtures in HTS, we explored a number of options. Our primary goal was to improve the quality of samples for HTS while minimizing the overall cost and required labor. First, we tested a solvent-solvent partitioning scheme with 216 extracts. Methanol extracts were filtered, dried and then partitioned between ethyl acetate and water. The three ethyl acetate layers were combined, dried, and subsequently partitioned between hexanes and methanol. In most cases, greater than 80% of the original extract remained in the aqueous layer. The aqueous layer was desalted by triturating with 1:1 ethyl acetate-MeOH, but the process was time consuming. In the end, the solvent-solvent partitioning protocol was labor intensive, time consuming, and used large quantities of solvents, but did not separate the organic components.

HPLC was considered too costly to use with crude extracts, not to mention the difficulty of obtaining sufficient material from an extract that is ~50-70% inorganic salt. Therefore, methods employing flash chromatography were explored. C_18_ Sep-Paks were tested, but did not have a high capacity compared to Diaion HP20SS. Diaion HP20SS, a porous polystyrene based adsorbent, was chosen as the solid support. Since HP20SS has large pores and a large surface area, it has high capacity to adsorb organic compounds. Additionally, the chromatographic behavior of HP20SS differs from C_18_ in that it also separates by size and has selectivity for aromatic compounds. Therefore, HP20SS represents an orthogonal chromatographic approach compared to C_18_. Initial studies indicated efficient desalting and separation of the organic components of each extract.

Prior to performing a large number of separations, tests were performed to standardize our extracts. Primarily, we hypothesized that there was little need to dry and weigh individual extracts prior to fractionation. Our hypothesis was based on the fact that the mixture of compounds present in each extract could not be predetermined; therefore, the concentration of individual components would be independent of the weight. Additionally, if the fractionation step concentrated organics, we would increase the hit rate above what we observed for pre-weighed extracts that were submitted to HTS.

We began by placing invertebrate samples (loosely packed) in a 120 mL polypropylene jar and covered with MeOH for a minimum of 24 hours. Then, 1 mL of extract was removed from 145 sponge and tunicate extracts. The solvent was removed, and the dried extract was weighed. We found that the average was 11.1 mg, and the standard deviation showed a narrow range (6.5 mg) with a minimum of 1.5 mg and a maximum of 36.9 mg. Additionally, we have observed that the majority of variation among extract weights was due to the amount of inorganic salts present in the crude extract. Overall, these results indicated that most samples could be processed without the need to weigh individual extracts.

Separation on HP20SS was investigated by varying the amount of HP20SS as well as the eluant used. Additionally, both wet-loading and dry-loading techniques were investigated. To test wet-loading, 10 mL of extract was filtered, and a slurry was prepared with 150 mg of HP20SS and loaded onto a plastic column. The solvent was forced through with a plastic syringe, the eluant diluted with 60 mL of H_2_O, and the column was reloaded with diluted sample to adsorb the organics onto the HP20SS. Subsequently, a 3-step elution (15 mL) was performed using 25% acetone/H_2_O, 50% acetone/H_2_O, and 100% acetone. The results were compared to a dry-loading procedure where the extract was placed on 150 mg of HP20SS, a slurry was prepared, and the solvent was removed using a centrifugal evaporator. The dried, “charged” resin was poured into a plastic column and subjected to a similar elution as the wet-loading method, but the first eluate (after optimization) was 15 mL of 100% H_2_O. In the end, the fastest method was to load an extract onto HP20SS (150 mg), dry the sample down in a centrifugal evaporator overnight, and then load the “charged” HP20SS into a column fitted with a frit. The extract was effectively desalted by washing the resin with 100% H_2_O. Subsequently, a four-step elution efficiently separated the organic components. The final optimized system utilized 100% H_2_O (FW), 25% IPA/H_2_O (F1), 50% IPA/H_2_O (F2), 75% IPA/H_2_O (F3), 100% MeOH (F4).

After the initial studies were performed, the second step was to develop a method that could be applied to a large number of samples. One of the goals was to prepare samples for screening without the need to weigh the extracts or the resulting fractions. Therefore, a pilot study was performed using 100 sponge extracts. The amount of material in each HP20SS fraction was subsequently weighed and the average calculated. Since the range from the sponge samples was tightly grouped, we could prepare all fractions in a similar manner without the need to obtain weights and provided an enormous time savings. To determine the effectiveness of the process, cytotoxicity for the crude extract was compared to the cytotoxicity of the four eluants (See Table 1) in HCT-116 cells using the MTT assay.

**Table 1:** Cytotoxicity results for crude extracts and HP20SS fractions.

Sample	Crude	Active Fraction
*Corticium* sp.	*^a^*NA	F1, F2
*Leucetta chagosensis*	NA	F3
Halichondriidae	NA	F2
*Xestospongia* sp.	NA	NA
Plakinidae	NA	F2, F3, F4
*Callyspongia* sp.	NA	F3
*Myrmekioderma* sp.	NA	F2, F3, F4
*Neopetrosia* sp. 1	NA	F2
*Neopetrosia* sp. 2	NA	F2
*Aaptos aaptos*	NA	F1
*Theonella swinhoei*	Active	F3, F4

*^a^*NA = Not Active

In most cases, we observed increased cytotoxicity in the HP20SS fractions compared to the crude extracts. For example, the *Corticium* sp. crude extract showed no cytotoxicity at 1.5 μg/mL, but F1 and F2 were found to be cytotoxic. F1 resulted in 15% survival at 2.4 μg/mL and F2 resulted in 12% survival at 1.8 μg/mL. Although the crude extract from *Theonella swinhoei* was cytotoxic, we observed increased cytotoxicity in the HP20SS fractions (See Table 2). For the two *Neopetrosia* spp., we observed consistent distribution of the activity. Overall, the HP20SS prefractionation strategy provided a cost-effective method to effectively desalt marine invertebrate extracts while providing an effective concentration of the organic components.

**Table 2:** Cytotoxicity results for *Theonella swinhoei*.

Sample	Concentration mg/mL	% Survival
Crude	3.4	78
FW	0.94	100
F1	7.5	100
F2	2.4	79
F3	3.5	6.4
F4	1.1	6.7

**Figure 1:**
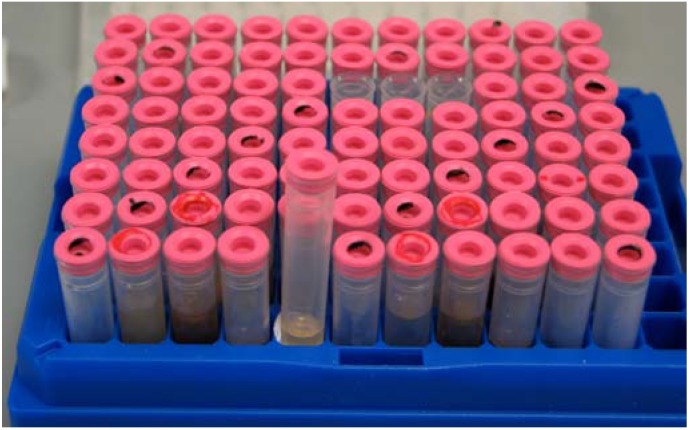
Example of HP20SS library storage.

Once extracts were fractionated, the HP20SS fractions were stored in 96-well format (10 mg/mL in DMSO) and daughter plates were made for screening. For all HP20SS fractions a material archive was maintained and stored at -80 °C in 96-well formatted polypropylene tubes (Figure 1).

We have been able to minimize the cost of the process by running eight columns in parallel in conjunction with metered solvent delivery from a one liter bottle. In terms of time, one person can process 16 samples in three hours using eight columns. Additionally, HP20SS can be regenerated and reused to minimize the overall cost. The best method we found for regenerating the HP20SS was to use a Soxhlet extractor with 1:1 dichloromethane:MeOH as an extraction solvent.

Our second goal was to expand our methodology to shorten discovery timelines by optimizing our dereplication strategy and increasing our throughput. Once hits were identified, we wanted to establish a rapid and automated approach to pursue hits. Therefore, we developed an automated LCMS fractionation protocol. We have utilized the same method to generate natural product libraries using a small number of marine invertebrates, and the potential of this approach for rapid drug discovery has been recently published [17]. By combining the HP20SS fractionation with an automated LCMS fractionation protocol, we generated high-purity libraries for screening and rapid drug discovery. The overall goal of the approach was to eliminate bioassay-guided isolation by identifying active compounds directly from the library using MS and NMR. This process was effectively demonstrated with a subset of the library in a BRCA2 phenotype-selective screen [17]. However, generating purified libraries with our entire annual collection was not a feasible option, but the method was used mainly to generate focused libraries for specific screening programs. Nonetheless, the method was designed to integrate with our HP20SS library.

Although purified libraries are attractive, there are distinct advantages to screening a prefractionated library compared to a purified natural products library [12,18]. The time line to structure identification is significantly shorter for purified natural products libraries, but screening a partially purified library allows more chemical diversity to be sampled and can limit the number of highly polar compounds that can interfere with assays. For most sponge and tunicate extracts, fewer highly lipophilic components are present, for example, compared to extracts from microbial fermentations. Nonetheless, rapid methods for identification and dereplication are necessary for many HTS programs. By using an automated LCMS fractionation protocol, components from the HP20SS library can be rapidly purified and characterized by accurate mass measurements to facilitate dereplication. Additionally, as screening capacity increases, partly through miniaturization of screening platforms, the potential to develop large high-purity natural product libraries becomes more attractive.

Once hits from the HP20SS library were identified, a 200 μL sample from the HP20SS material archive was subjected to the LCMS fractionation protocol. The LCMS fractionation utilized a Q-tof micro mass spectrometer equipped with lockspray to enable accurate mass measurements. The effluent from the HPLC was directed into a splitter where a portion of the sample was infused into the Q-tof while most was directed into a 96-well collection plate. The collection time for each well was mapped onto the chromatogram using FractionLynx. After fractionation the contents of the collection plate could be split, one plate for screening and one plate for a material archive and NMR. As previously noted [17], the key to this separation strategy was the use of a Phenomenex 3 mm ° 100 mm Onyx^TM^ C18 monolithic HPLC column. The monolithic column has higher capacity compared to a traditional HPLC column [19] and has easily handled injections of HP20SS fractions (2 mg) dissolved in 200 μL of DMSO. Although we have successfully used this approach on numerous hits, two examples will be presented here.

The approach was applied to a project where an HP20SS fraction from *Pseudoceratina purpurea* was identified as a hit in a luciferase assay. A sample of the active fraction from the archive was subjected to the automated LCMS fractionation protocol. The contents of the collection plate were split with a portion being submitted for assay and the remaining portion for a material archive. The most active well (~50 μg) was analyzed by NMR and allowed rapid identification of the active compound as psammaplin A. The details of the assay and additional work have been published elsewhere [20]. In the end, the identification of the principal active component was achieved in less than 24 hours after the assay results were obtained on the LCMS fractions. However, it should be noted that on such a small scale, NMR analysis required a cryo probe operating at 600 MHz. The example demonstrates the speed at which active compounds can be identified by integrating a partially purified library with automated LCMS fractionation.


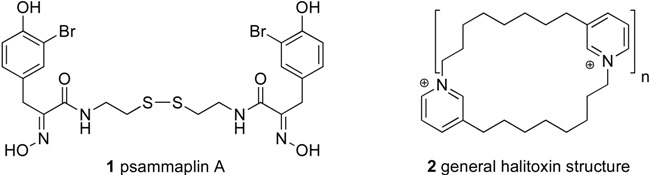


**Figure 2:**
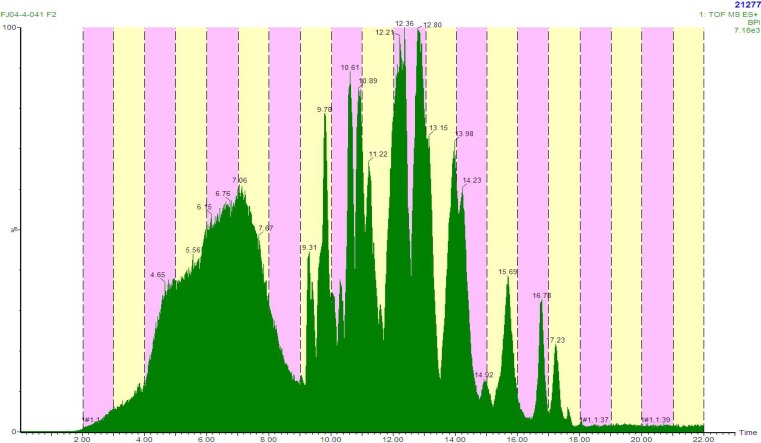
LCMS chromatogram showing effective separation of pyridinium polymers.

Additionally, we have utilized this methodology to rapidly identify non-selective inhibitors. An active HP20SS fraction was identified as a hit in a HT screen, but based on field collection notes most likely contained alkyl pyridinium polymers, which have commonly hit in HTS. Therefore, we subjected an HP20SS sample to LCMS fractionation in an attempt to separate the pyridinium polymers from other potentially active natural products. The chromatogram showed a broad poorly defined peak eluting between three minutes and nine minutes (Figure 2). The mass spectra in this region of the chromatogram showed prominent ions that are consistent with halitoxin-like fragments at *m/z* 379.3123 (calcd for C_26_H_39_N_2_, 379.3113) and *m/z* 190.1585 (calcd for C_13_H_20_N, 190.1596) [21]. Overall, we could utilize the monolithic column to effectively elute the pyridinium polymers early in the chromatography with the subsequent separation of other components (Figure 2). The activity was correlated to the wells that exhibited halitoxin-like fragments in the mass spectra, and the project was quickly dropped.

With respect to creating high-purity natural product libraries, application of HPLC in conjunction with mass spectrometry and evaporative light scattering detection was first demonstrated using plant extracts [22,23]. Although the potential of natural products as a source of pharmaceutical leads has clearly been demonstrated [24,25], a number of technical concerns have surfaced regarding natural product libraries. Libraries containing only pure compounds are costly and take more time to produce, but are more amenable to the high throughput paradigm due to decreased assay interference and the potential rapid structure identification of assay hits. As previously stated, the drawback to purified compound libraries is that less chemical diversity is sampled. Prior to generating a high-purity natural products library, several technical concerns must be addressed. For example, the method of purification, in part, must be automated. Since the size of a library is likely to be large, library storage and sample handling is a concern. The quantity of material in the library is another concern. Since modern spectroscopic instrumentation has become increasingly sensitive and modern assays require only small amounts of material, a high-purity library can be constructed and only contain small quantities (50 μg to 1 mg) of material. However, quantitation becomes difficult with microgram quantities, but can be addressed using ELS detection [26,27], CA detection [27], or by NMR [28]. Finally, data management quickly becomes difficult without an appropriate database.

**Figure 3:**
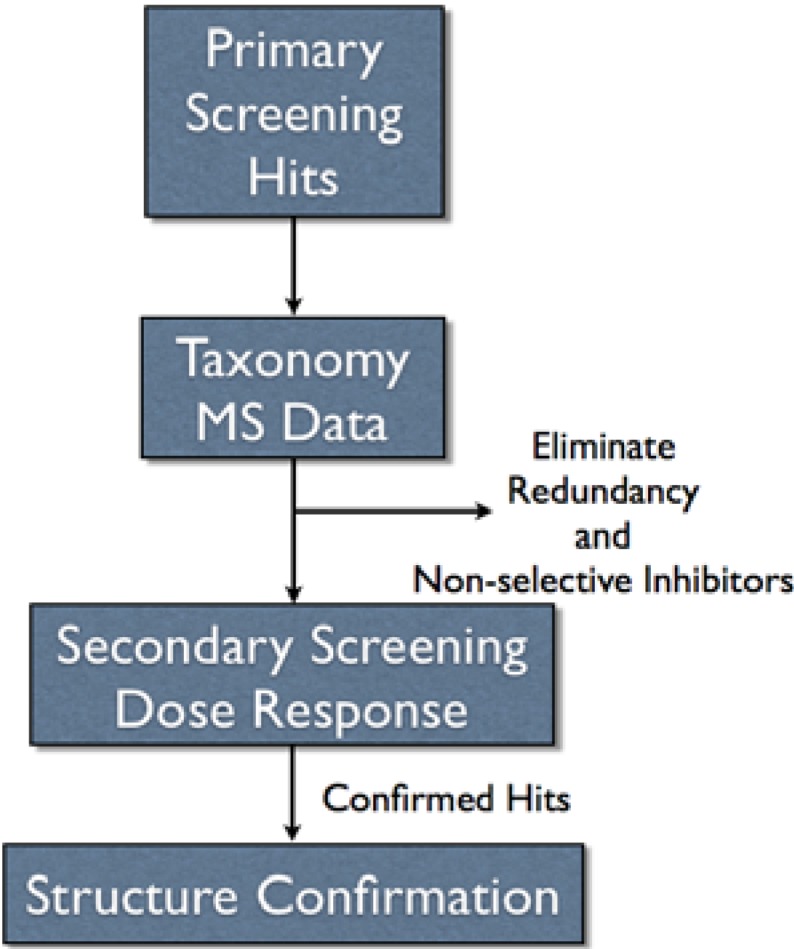
Overall scheme for pursuing assay hits.

Our LCMS fractionated library was constructed by assuming equal distribution of material among 80 wells in a collection plate with each collection plate representing one marine invertebrate. The drawback to assuming equal distribution was that some wells contained little or no material while other wells contained larger quantities of material. Another issue that surfaced was data management. We decided to obtain accurate mass measurements on analytes as the purification proceeded. The MS data provided a mechanism to track the contents of the library, but the raw data files from a Q-tof were large (~250 mb per HPLC run). However, we were able to process the data “on-the-fly” providing lock mass corrected, centroided accurate mass data. Although some information was lost, the data files were reduced to ten to fifteen mb per run allowing easy automated backup of data without the need for a large data server. Obtaining the MS data during fractionation was advantageous since it did not require retrieval of plates from storage and subsequent LCMS analysis for prioritization of hits and dereplication (Figure 3), which would add significant complication and require more time. Additionally, having the MS data in conjunction with taxonomy provided a mechanism to remove compound redundancy and known non-selective inhibitors prior to secondary screening (Figure 3).

Overall, this effectively streamlined the process of prioritizing hits from primary screening. Plates were only removed from storage once a hit had been confirmed and yielded an appropriate dose response curve. Then, samples were removed from storage and analyzed by NMR. Overall, the method provided libraries and methods that were compatible with HTS timelines.

Recently, we began attempting to scale up the procedure in an attempt to provide more material for screening and structure elucidation. Utilizing an HP20SS fraction from a *Pipestela* sp. that contained large quantities of milnamide A (**3**) and milnamide D (**4**), the capacity of a 4.6 ° 100 mm monolithic C18 column was compared to that of the 3 ° 100 mm column.


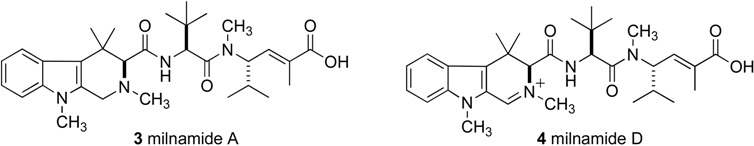


**Figure 4:**
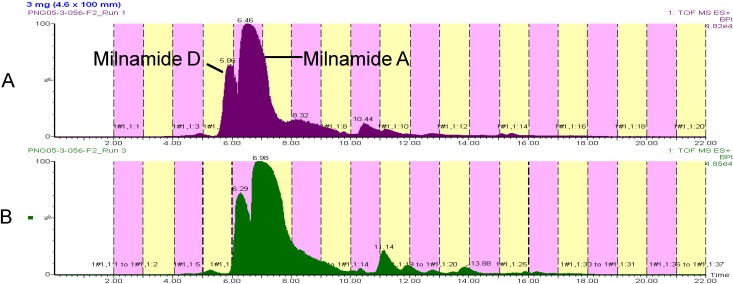
Comparison of chromatography on a 3 mm ID column (A) and a 4.6 ID column (B).

Since flow rates were ultimately a function of our collection volume, a flow of 2 mL/min could not be exceeded even though the scaled flow from 3 mm ID (1.5 mL/min) to 4.6 mm ID would be 3.53 mL/min. Nonetheless, a 50% increase in loaded material for the 4.6 mm ID column, from 2 mg to 3 mg, yielded similar resolution and only a slight change in retention time (See Figure 4). Overall, the use of a 4.6 mm ID column will increase the final amount of material in the natural product library, without the need to change the collection strategy from 96-well format.

## Conclusions

In conclusion, marine invertebrate extracts can be rapidly separated on HP20SS. The method effectively separates the organic constituents from the inorganic salts and can concentrate the active principal components. By creating partially purified libraries, daughter plates can be easily generated for new screening programs. Additionally, identifying compounds directly from the LCMS fractionated material archive facilitates rapid dereplication and purification of active components as compared to returning to the source organism. Integrating the HP20SS library with our automated LCMS fractionation protocol has increased the number of projects that we can pursue as well as allowing rapid dereplication.

An additional outcome of our work on HP20SS fractionation has been that the method was widely applicable. The potential for variation in chemistry among sponge and tunicate species due to variations in microbial populations, seasonal, or environmental changes does exist, and the chemistry within one species is likely to show some variation. However, variability should increase the chemical diversity of the library, and variability can be easier to identify with partially purified extracts as compared to crude extracts.

## Experimental

### General

All NMR data were obtained at 600 MHz on a Varian INOVA equipped with a cryogenically cooled ^1^H channel. For gCOSY, gHMBC, gHSQC experiments, standard vendor supplied pulse sequences were used. Samples were dissolved in 250 μL of DMSO-*d*_6_ (Cambridge Isotope Labs) and placed in a 5 mm, DMSO matched Shigemi tube. Spectra were referenced to DMSO resonances (δ_H_ 2.49 ppm; δ_C_ 39.5 ppm). All solvents used for HPLC were either HPLC or Optima LCMS grade (Fisher Scientific). HP20SS was purchased from Sigma.

### Biological Material.

The sponge *Pseudoceratina purpurea* (Carter, 1880) was collected by SCUBA off the coast of Fiji in 2004 (S 17º 00.064’, E 178º 37.705’). A voucher specimen (FJ04-4-36) is retained at the University of Utah.

### Extraction and Chromatography

All invertebrates were extracted with MeOH. The MeOH extract (24 mL) was loaded onto HP20SS (300 mg) and subsequently dried using a centrifugal evaporator. After the sample was dried, the “loaded” HP20SS was transferred to a 2.5 ° 9 cm polypropylene column fitted with a frit. Fifteen mL of each solvent (100% H_2_O, 25% IPA/H_2_O, 50% IPA/H_2_O, 75% IPA/H_2_O, and 100% MeOH) were pushed through the sample using a 60 mL syringe fitted to the top of the column to generate five eluates (FW, F1, F2, F3, F4, respectively). The solvents were removed from all samples using a centrifugal evaporator.

### LCMS Fractionation

Experimental details are the same as those previously published [17].

### LCMS Fractionation of Psammaplin A Containing Sample

The LCMS fractionation of the active HP20SS fraction (4WA7 = FJ04-4-36-F2), utilized a sample of archive material (200 mL, 5.0 mg/mL) and was subjected to the fractionation scheme. The active fraction was number six and eluted between eight and nine min. MS gave [M+H]^+^*m/z* 662.9578, calcd. for psammaplin A (**1**) C_22_H_25_^79^Br_2_N_4_O_6_S_2_, 662.9582.
